# Efficacy and safety of expanded hemodialysis in hemodialysis patients: a meta-analysis and systematic review

**DOI:** 10.1080/0886022X.2022.2048855

**Published:** 2022-03-28

**Authors:** Yuchao Zhao, Liangying Gan, Qingyu Niu, Mengfan Ni, Li Zuo

**Affiliations:** Department of Nephrology, Peking University People’s Hospital, Beijing, P. R. China

**Keywords:** Albumin, expanded hemodialysis, uremic toxin, meta-analysis

## Abstract

**Background:**

Expanded hemodialysis (HDx) is a new dialysis modality, but a systematic review of the clinical effects of using HDx is lacking. This systematic review and meta-analysis aimed to assess the efficacy and safety of HDx for hemodialysis (HD) patients.

**Methods:**

PubMed, the Cochrane library, and EMBASE databases were systematically searched for prospective interventional studies comparing the efficacy and safety of HDx with those of high flux HD or HDF in HD patients.

**Results:**

Eighteen trials including a total of 853 HD patients were enrolled. HDx increased the reduction ratio (RR) of β2-microglobulin (SMD 6.28%, 95% CI 0.83, 1.73, *p* = .02), κFLC (SMD 15.86%, 95% CI 6.96, 24.76, *p* = .0005), and λFLC (SMD 22.42%, 95% CI, 17.95, 26.88, *p* < .0001) compared with high flux HD. The RR of β2-microglobulin in the HDx group was lower than that in the HDF group (SMD −3.53%, 95% CI −1.16, −1.9, *p* < .0001). HDx increased the RRs of κFLC (SMD 1.34%, 95% CI 0.52, 2.16, *p* = .001) and λFLC (SMD 7.28%, 95% CI 1.08, 13.48, *p* = .02) compared to HDF. There was no significant difference in albumin loss into the dialysate between the HDx and HDF groups (SMD 0.35 g/session, 95% CI −2.38, 3.09, *p* = .8).

**Conclusions:**

This meta-analysis indicated that compared with high-flux HD and HDF, HDx can increase the clearance of medium and large-molecular-weight uremic toxins. And it does not increase the loss of albumin compared with HDF.

## Introduction

The number of end-stage kidney disease (ESKD) patients is increasing around the world, and the number of patients undergoing hemodialysis (HD) is continuously increasing [1]. ESKD induces retention of uremic toxins. These toxins can be grouped into small molecules (<500 Da), middle-sized molecules (>500 Da–60 kDa), and protein-bound molecules [2]. Low-flux HD is a classical HD treatment that can effectively remove small molecular toxins through diffusion, but the elimination of middle-sized molecules is poor [3]. The retention of middle-sized molecules is related to increased mortality in HD patients [4].

To enhance the elimination of middle to large-sized molecular toxins, new treatments, such as high-flux dialysis and hemodiafiltration (HDF) have been applied in clinical practice. High-flux HD enables the elimination of small to middle-sized molecules, but it is not sufficient for removing molecules larger than 15 kDa [5]. These larger molecules, such as κFLC and λFLC, are associated with disease progression and mortality in HD patients [6]. High serum FLC levels increased risk of vascular calcification, ESKD progression, inflammation, and levels of other uremic toxins and increased risk of mortality [6]. Online HDF increased the clearance of middle-sized molecular toxins compared to high-flux HD [7]. The use of this technology has improved the prognosis of ERSD patients compared with both low-flux and high-flux HD, but the application of HDF is limited by its relatively higher cost and the more complex nature of the technique [7]. High cutoff membranes can be used to enhance the removal of middle-sized molecules, but lead to hypoalbuminemia [8]. Medium cutoff (MCO) dialyzers, also known as high retention onset membranes, are a novel class of dialyzers that can clear middle-sized and large uremic toxins close to the molecular size of albumin [9,10].

Expanded hemodialysis (HDx) is a new kind of HD that uses an MCO dialyzer. Several studies have demonstrated the efficacy and safety of HDx in HD patients [7–10]. Nevertheless, a lack of systematic evaluation limits the use of HDx in clinical practice. We therefore conducted a systematic review and meta-analysis to evaluate the efficiency of HDx for the removal of small molecular toxins and middle to large-sized molecular toxins such as β2-microglobulin, κFLC, and λFLC, and assess changes in serum albumin levels.

## Methods

### Search strategy

A systematic review was performed by searching PubMed, the Cochrane Library, and Embase databases for relevant studies published from inception to 19 May 2021. The search language was limited to English. The following search terms were used in PubMed and were changed depending on the rules of each database:

(HDx) OR (Expanded HD) OR (HDx) OR (Medium cut off) OR (Mid cutoff) OR (Medium-cut off) OR (Mid-cut off) OR (MCO) OR (Mid cutoff) OR (MCO-HD) OR (MCO HD) OR (Theranova).
(HD OR Renal Dialysis [MeSH Terms]) OR (HD) OR (Dialyzer) OR (Dialyzer) OR (Membrane).
1 AND 2.

### Protocol and registration

No registered protocol.

### Selection criteria

Trials were selected with the following eligibility criteria: (1) Prospective interventional studies that enrolled ESKD patients undergoing HD; (2) The experimental group was HDx; (3) Controls were standard HD or HDF; (4) One or more outcomes of interest were reported, including Kt/V, reduction ratio (RR) of *β*2-microglobulin, RR of κFLC, RR of λFLC, change in predialysis serum albumin, and albumin loss in dialysate. Clinical studies with the following features were excluded: (1) Published in the form of letters, case reports, comments, or conference abstracts; (2) Retrospective studies; (3) Data were unavailable to calculate standardized mean differences (SMDs) or odds ratio (OR).

### Data extraction and quality assessment

Two investigators (ZYC and GLY) extracted the data from enrolled studies independently. Disagreements between investigators were resolved by consensus. A kappa statistic calculated for measuring agreement between two authors during the systematic searches. The kappa of agreement was 0.892. The data collected from the selected studies included the first author and publication year, background treatment in both study groups, types of intervention, study duration, patient ages, and relevant outcomes (Kt/V, RR of β2-microglobulin, RR of κFLC, RR of λFLC, change in predialysis serum albumin, and albumin loss in dialysate).

### Risk of bias assessment

The risk of bias was assessed by two authors using the Cochrane risk of bias tool independently [11]. The trials enrolled were assessed and graded as low, unclear, or high risk.

### Data synthesis and analysis

Standard mean differences (SMDs) in outcomes and 95% confidence intervals (CIs) were calculated as effect measures. The Mantel–Haenszel *χ*^2^-based test and *I*^2^ test were used to evaluate heterogeneity among randomized controlled trials. A fixed-effects model was used if heterogeneity was <50%, whereas a random-effects model was used if heterogeneity was ≥50%. Subgroup analysis was performed based on different treatments in control groups. Sensitivity analysis was performed by excluding any single study. Publication bias was tested by funnel plots. The meta-analyses were conducted using Revman version 5.3 software .

## Results

### Study characteristics

**Figure 1.**
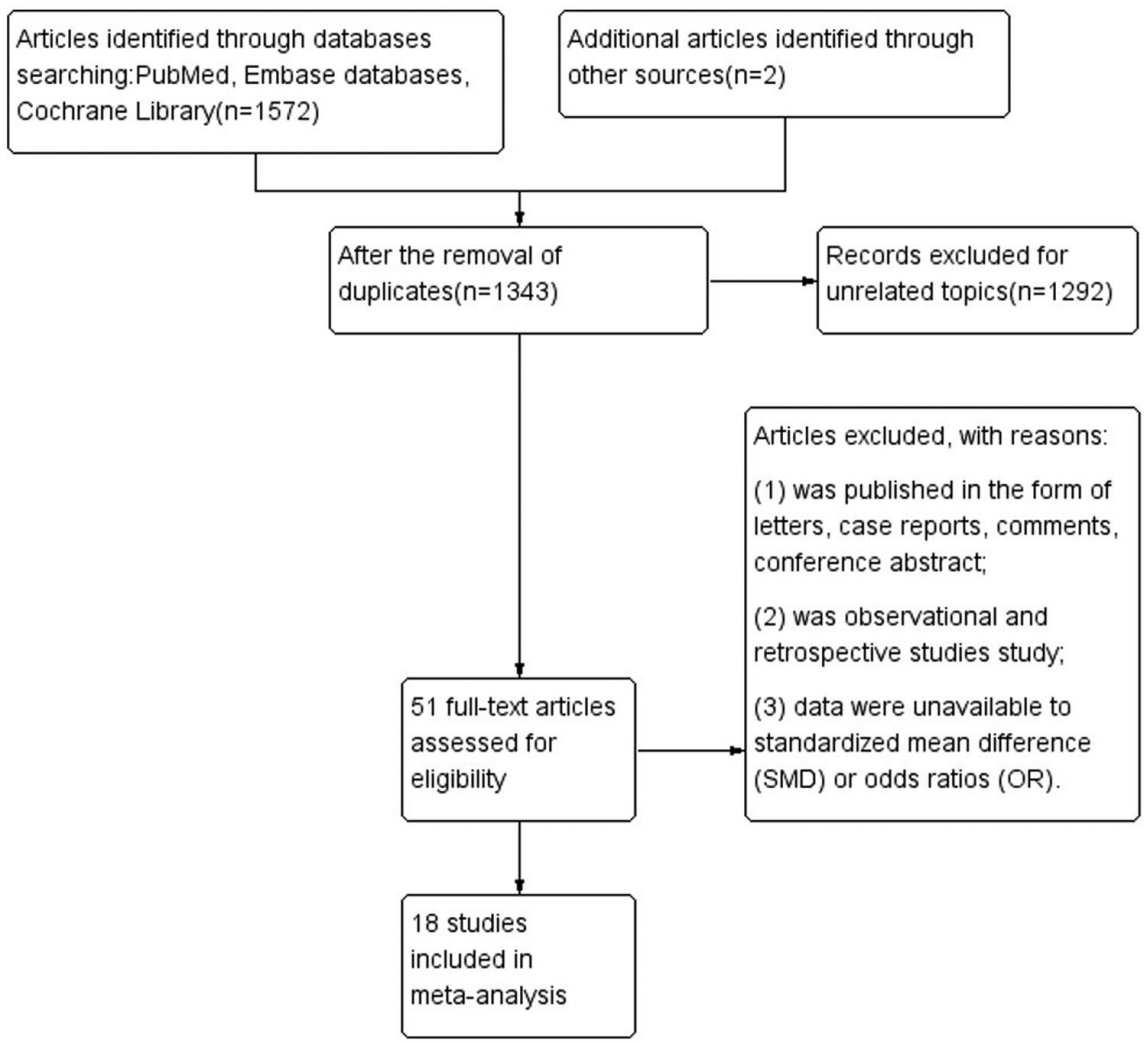
The entire search strategy.

The entire search strategy is illustrated in Figure 1. The search identified 1572 articles, 229 of which were excluded as duplicates, then 1343 titles were screened, and 51 full-text articles were assessed. Eighteen eligible studies including a total of 853 participants were used to evaluate the efficacy and safety of HDx. Characteristics of the 18 studies included are presented in Table 1.

**Table 1.** Characteristics of the included studies in the meta-analysis.

Study ID	Location	Treatment	Controls	Study duration (months)	Male, No. (%)	Age
Kirsch et al. [1]	Germany	HDx	HDF	–	16 (80)	65.4
Kirsch et al. [1]	Austria	HDx	HF-HD	–	12 (63.2)	55.4
Zickler et al. [12]	Germany	HDx	HF-HD	3	35 (72.9)	58.1
Reque et al. [7]	Spain	HDx	HDF	2	3 (37.5)	69
García-Prieto et al. [13]	Spain	HDx	HD/HDF	1/4	9 (50)	65
Maduell et al. [14]	Spain	HDx	HF-HD/HDF	–	17 (80.95)	63.2
Maduell et al. [15]	Spain	HDx	HDF	–	16 (76.2)	65.4
Kim et al. [16]	Korea	HDx	HD/HDF	1/4	6 (100)	66.1
Cho et al. [17]	Korea	HDx	HF-HD	12	33 (57.9)	54.6
Arrascue et al. [18]	Spain	HDx	HDF	6	28 (65.1)	61.3
Cordeiro et al. [19]	Brazil	HDx	HDF	1	11 (69)	40.7
Sevinc et al. [10]	其					
	Turkey	HDx	HF-HD	3	29 (48)	56.4
Weiner et al. [20]	America	HDx	HF-HD	6	105 (61)	59
Lim et al. [21]	Korea	HDx	HF-HD	3	33 (67.3)	62.2
Belmouaz et al. [22]	France	HDx	HF-HD	3	28 (70)	75.5
Yeter et al. [5]	Turkey	HDx	HD	6	26 (63)	52.9
Lindgren et al. [23]	Sweden	HDx	HDF	–	–	59.6
Cozzolino et al. [8]	Italy	HDx	HF-HD	3	16 (76)	71

### Evaluation of the risk of bias

**Figure 2.**
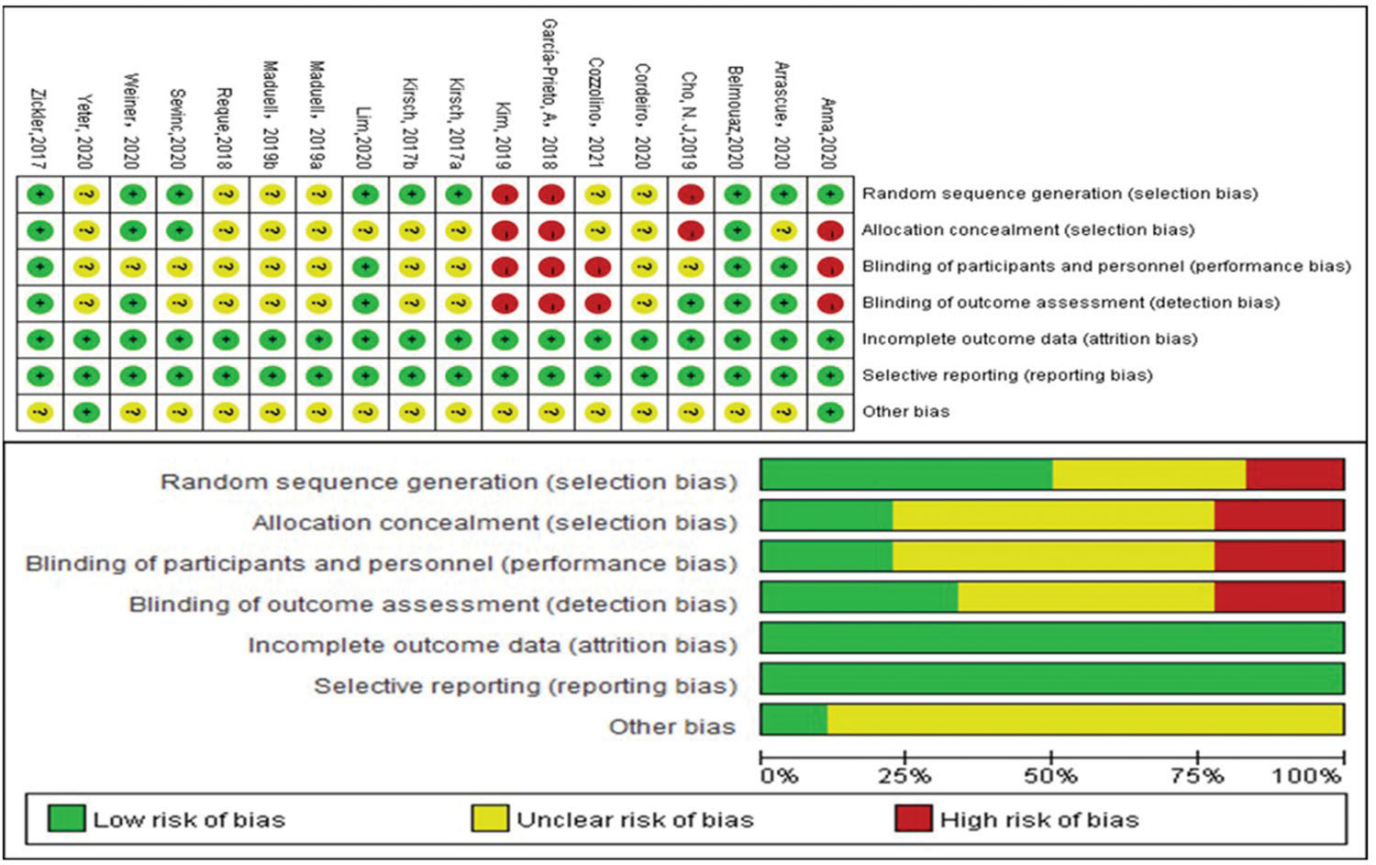
The risk of bias assessment.

The risk of bias in the studies was assessed using the Cochrane Risk-of-Bias tool. The risk of bias assessment is shown in Figure 2. Overall studies had a low risk of bias. Eight studies described adequate randomization, and three studies had a high risk of bias with respect to randomization. Others did not describe the sequence generation methodology. Allocation concealment and blinding of the outcome were unclear in most studies because detailed information was not provided. Incomplete outcomes and selective reporting were at low risk of bias in all of the studies. With respect to other bias, most studies were determined to be at unclear risk.

### Efficacy assessment

#### Kt/V

**Figure 3.**
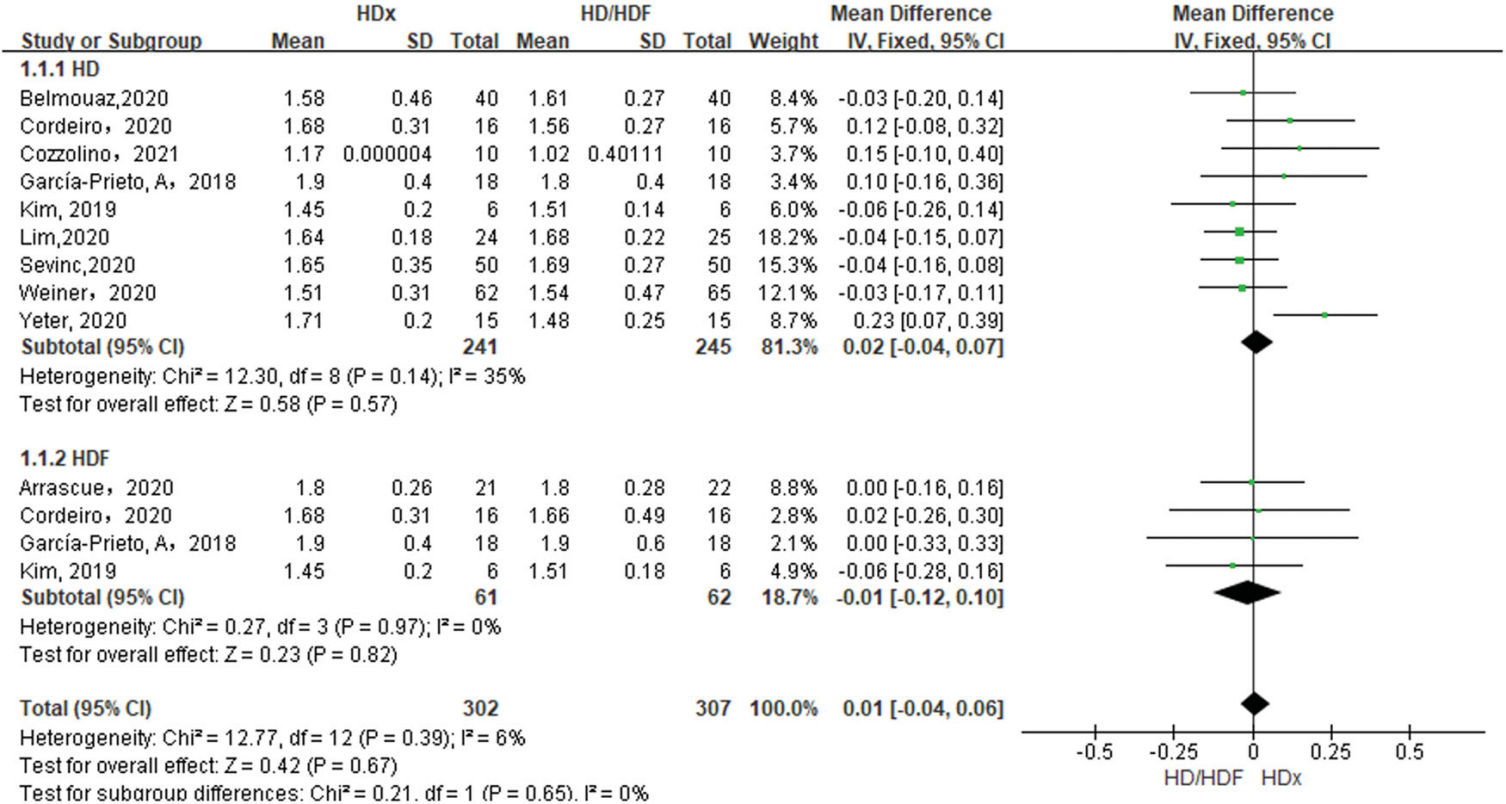
Forest plot for Kt/v.

Single-pool Kt/V was the primary outcome of this meta-analysis, and nine studies that included a total of 486 participants compared the Kt/V associated with HDx and HD. Four studies that included a total of 123 participants compared the Kt/V associated with HDx versus HDF. There were no significant differences in Kt/V between HDx and HD or HDF (SMD 0.02, 95% CI −0.04, −0.07, *p* = .57 and SMD −0.01, 95% CI −0.04, 0.06, *p* = .82, respectively) (Figure 3).

### Reduction rate of *β*2-microglobulin

**Figure 4.**
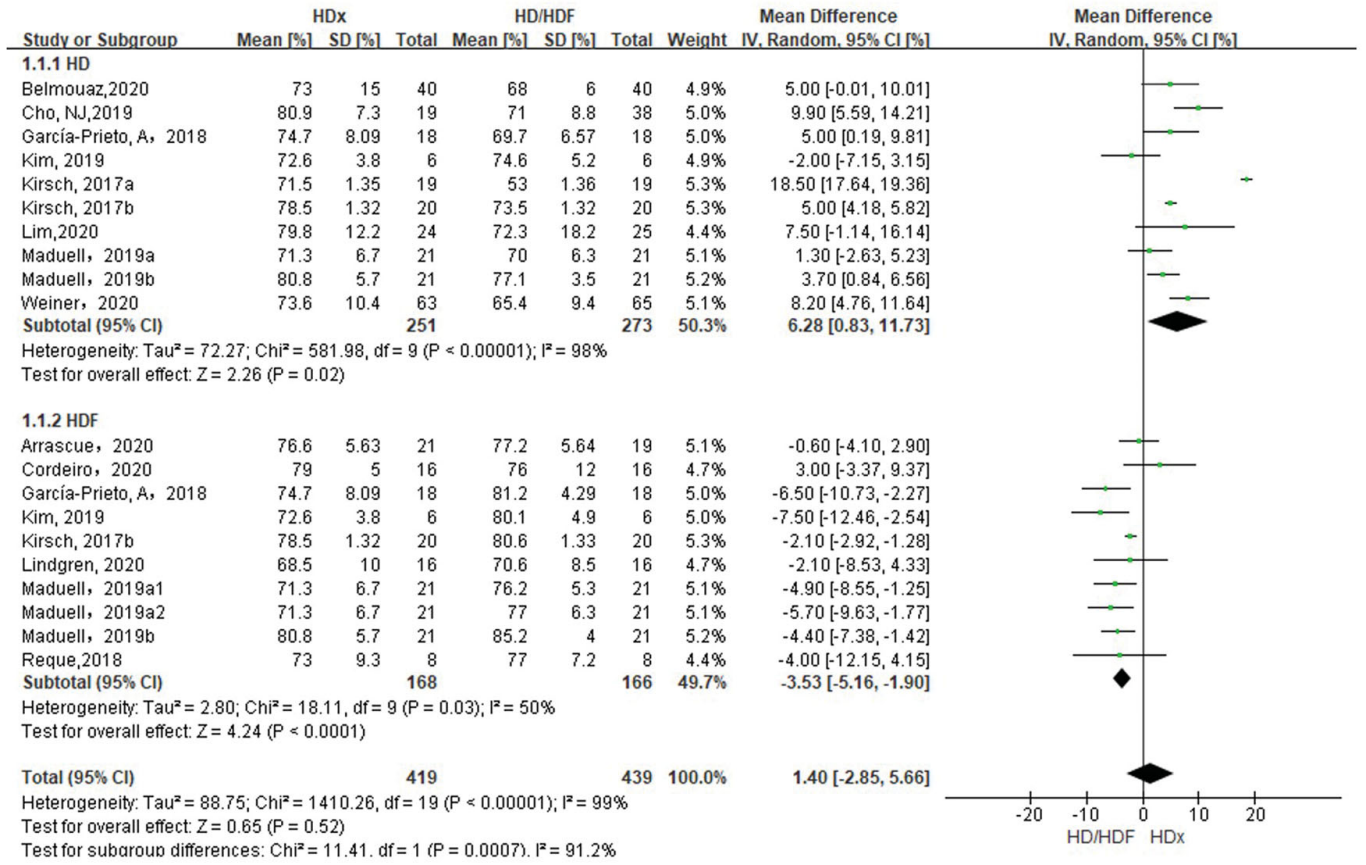
Forest plot for reduction rate of *β*2-microglobulin.

The RR of *β*2-microglobulin in the HDx group was significantly higher than that in the HD group (SMD 6.28%, 95% CI 0.83, 11.73, *p* = .02), but lower than that in HDF group (SMD −3.53%, 95% CI −5.16, −1.9, *p* < .0001) (Figure 4).

### Reduction rate of κFLC

**Figure 5.**
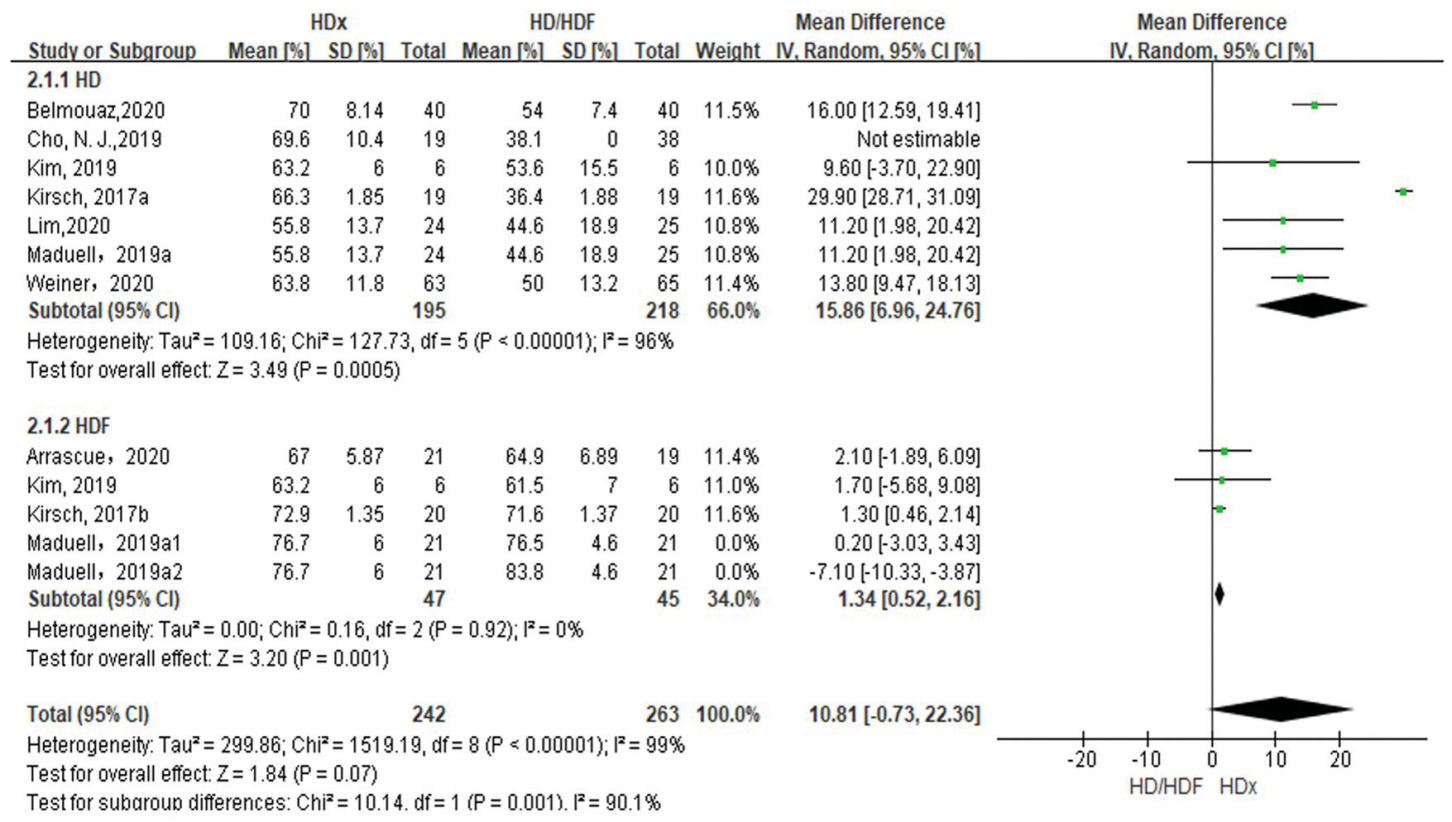
Forest plot for reduction rate of κFLC.

The results of meta-analysis comparing the RR of κFLC in HDx with those of HD and HDF are shown in Figure 5. The RR of κFLC was significantly higher in HDx than in HD and HDF (SMD 15.86%, 95% CI 6.96, 24.76, *p* = .0005 and SMD 1.34%, 95% CI 0.52, 2.16, *p* = .001, respectively) (Figure 5).

### Reduction rate of λFLC

**Figure 6.**
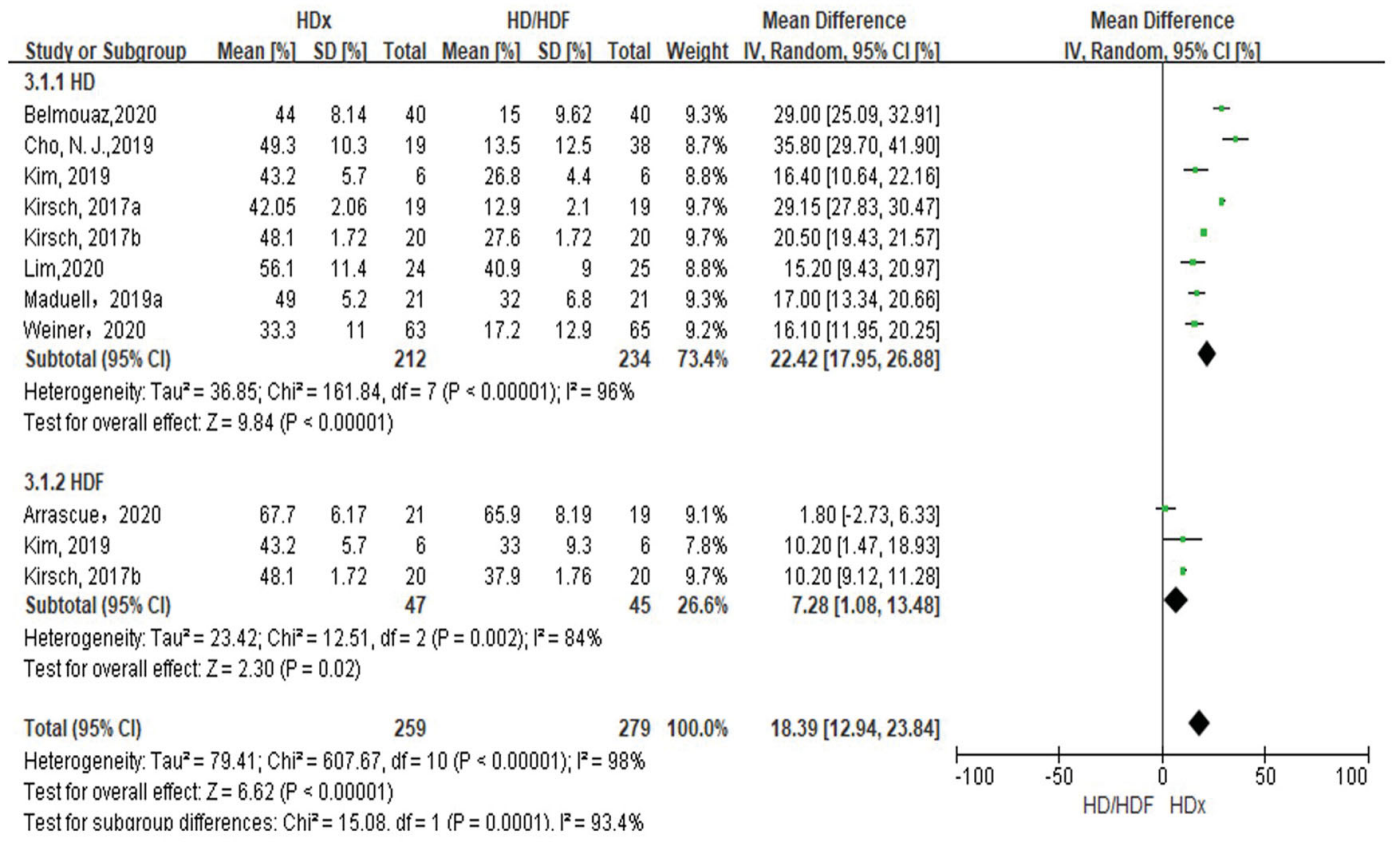
Forest plot for reduction rate of λFLC.

The RR of λFLC was examined in 11 trials. HDx significantly increased the RR of λFLC compared with HD (SMD 22.42%, 95% CI 17.95, 26.88, *p* = .0001). The RR of λFLC in HDx was significantly higher than that of HDF (SMD 7.28%, 95% CI 1.08, 13.48, *p* = .02) (Figure 6).

### Safety assessments

#### Predialysis serum level of albumin

**Figure 7.**
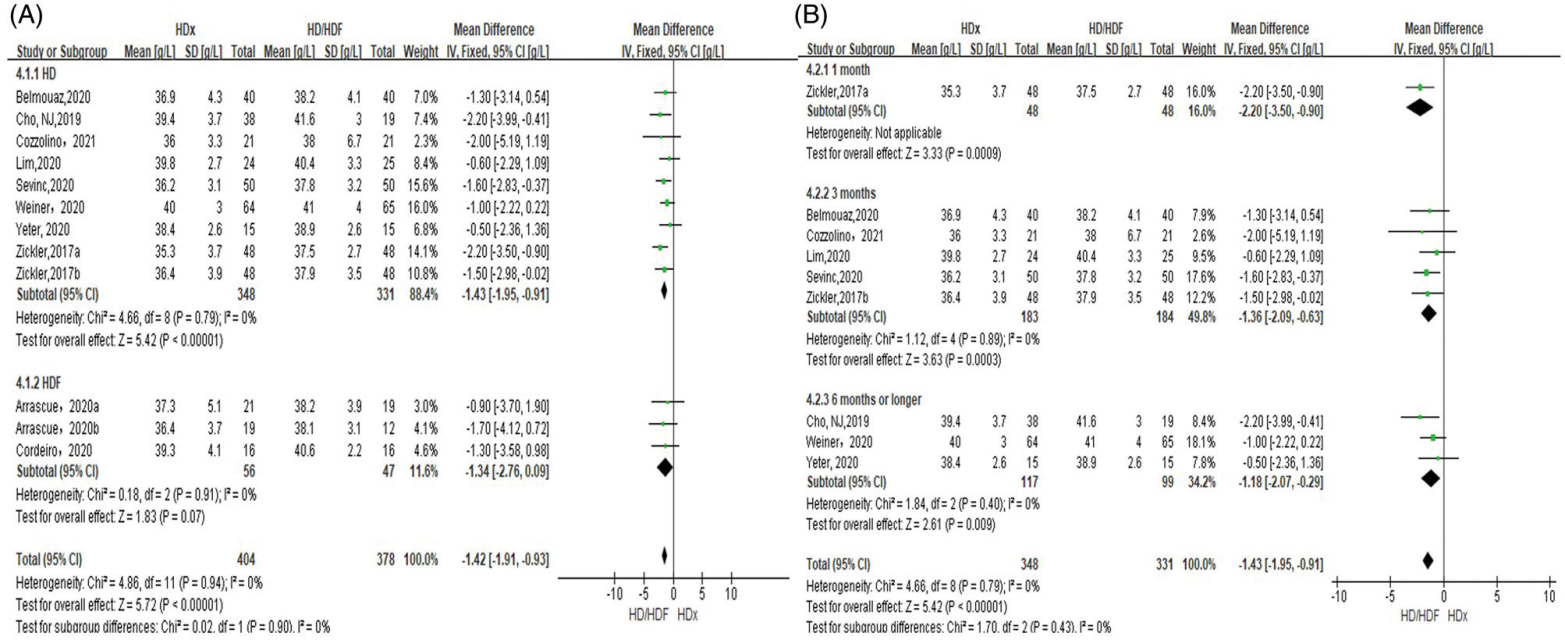
Forest plot for predialysis serum level of albumin.

Predialysis levels of serum albumin were significantly lower in the HDx group than in the HD group (SMD −1.43 g/L, 95% CI −1.95, −0.91, *p* < .00001) (Figure 7(A)). There was no significant difference in predialysis serum albumin levels between the HDx group and the HDF group (SMD −1.34 g/L, 95% CI −2.76, 0.09, *p* = .07). Zickler et al. [12] measured predialysis serum albumin after 1 month of treatment; Belmouaz et al. [22], Mario Cozzolino et al. [24], Lim et al. [25], Sevinc et al. [10], and Zickler et al. [12] measured predialysis serum albumin after 3 months of treatment; Weiner et al. [20], Yeter et al. [5], and Cho et al. [17] measured predialysis serum albumin after ≥ 6 months. In subgroup analysis (Figure 7(B)), with the extension of follow-up time the difference in predialysis albumin level between the HDx and HD groups gradually decreased (1 month SMD −2.2 g/L, 95% CI −3.5 to −0.9, *p* = .0009, 3 months SMD −1.36 g/L, 95% CI −2.09, −0.63, *p* = .0003, ≥6 months SMD −1.18 g/L, 95% CI −2.07, −0.29, *p* = .009).

#### Albumin loss in the dialysate

**Figure 8.**
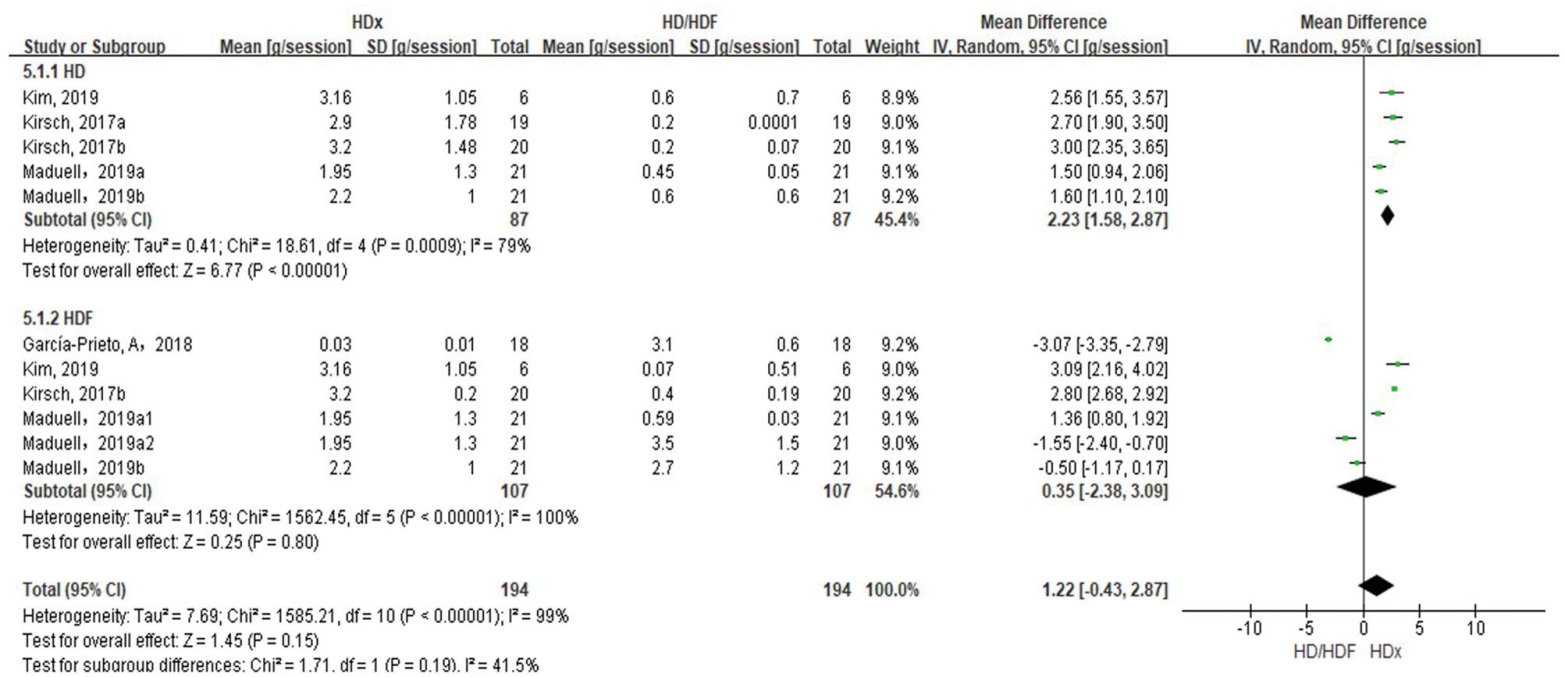
Albumin loss in the dialysate.

Six studies compared albumin loss in the dialysate for HDx *versus* HD and HDF (Figure 8). Albumin loss in the dialysate in the HDx group was significantly higher than that in the HD group (SMD 2.23 g/session, 95% CI 1.58, 2.87, *p* < .00001). There was no significant difference in albumin loss in the dialysate between the HDx and HDF groups (SMD 0.35 g/session, 95% CI −2.38, 3.09, *p* = .8). In a trial reported by García-Prieto et al. [13], albumin loss in the HDx group was significantly different to that in other trials. In García-Prieto’s trial [13], HDF was performed using an FX CorDiax 1000 dialyzer, of which the effective surface area and UF coefficient are significantly higher than those of the HDx group of dialyzers (2.3 versus 1.7–2.0 m^2^, 68 versus 48–59 mL/h/mmHg). It is unclear whether ultrafiltration was considered. A sensitivity analysis was performed that separately excluded this trial, and the results remained unchanged (Supplemental Figure 1). In order to reduce the influence of residual kidney function on pre-dialysis serum levels of albumin, we further compared residual kidney function in related studies. The results showed that there was no difference in residual kidney function between HDx group and HD or HDF group (Supplemental Table 2).

#### Adverse events (AEs)

**Figure 9.**
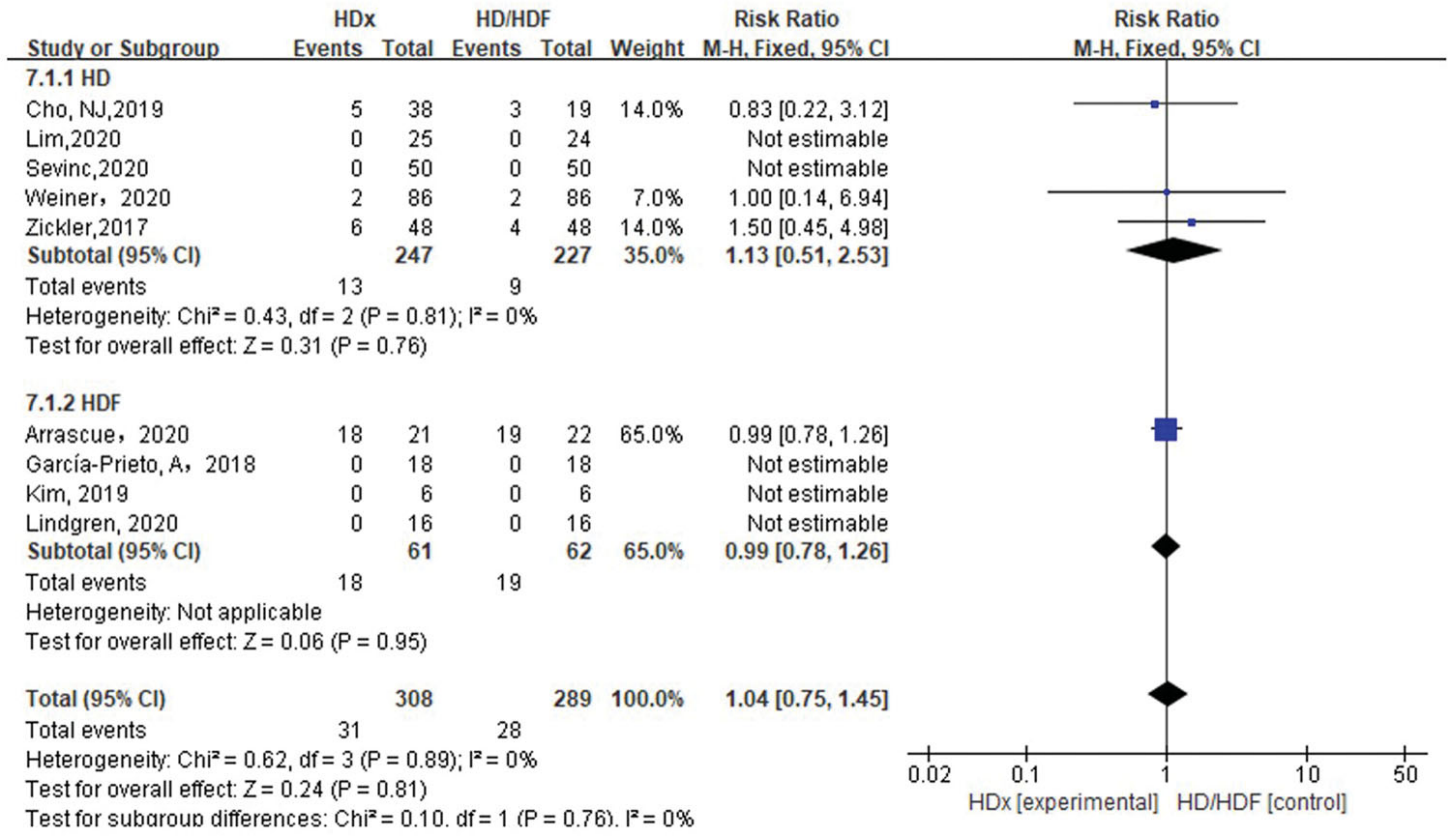
Forest plot for AEs.

We added adverse events (AEs) as safety outcomes in our meta-analysis. As depicted in Figure 9. There was no difference between HDx and HD or HDF in the incidence of AEs (1.12, 95% CI [0.50, 2.52], *p* = .78; 0.99, 95% CI [0.78,1.26], *p* = .95).

## Discussion

The current meta-analysis provides evidence for the efficacy and safety of HDx in HD patients. There was no significant difference in Kt/V between HDx and HD or HDF. The RRs of β2-microglobulin, κFLC, and λFLC were significantly greater in the HDx group than in the HD group. HDx is less effective for removing β2-microglobulin than HDF, and with respect to larger molecular toxins, such as κFLC and λFLC, removal was better in the HDx group than in the HDF group. Albumin loss in the HDx group was significantly greater than that in the HD group, but comparable to that in the HDF group.

Patients with ESKD have a higher mortality rate, which is related to the accumulation of uremic toxins. Uremic toxins are grouped into small (<500 Da), middle-sized (>500 Da) molecular water-soluble solutes, and protein-bound substances [1]. Small molecular toxins such as urea can be effectively removed by traditional HD, but middle-sized molecular toxins, such as *β*2-microglobulin (11.8 kDa), κFLC (22.5 kDa), and λFLC (44.5 kDa) are poorly removed by conventional HD modalities. Studies have confirmed that middle-sized molecules are associated with a poor prognosis in dialysis patients [1]. A number of studies indicate that serum *β*2-microglobulin is a predictor of cardiovascular events, including myocardial infarction, heart failure, and stroke [26,27]. In ESKD patients, poor renal clearance leads to increased levels of serum κFLC and λFLC. Trials suggest that elevated serum FLC is an independent risk factor for mortality in ESKD patients [6,28]. To improve the prognosis of CKD patients, the treatment mode of HDF have been used to increase the clearance of middle-sized molecular toxins, but the application of HDF is limited by its cost and the complex nature of the technique. HDx therapy is a novel modality that incorporates an MCO membrane applied in HD mode [9]. The newest generation of MCO membranes enables the removal of large molecules up to a molecular weight of 45 kDa, with a sieving coefficient for albumin of 0.008 [24] which limits albumin loss [23]. The present meta-analysis indicates that compared with HD, HDx has the same ability to remove small molecular toxins but it can remove medium-sized and large molecular toxins more effectively. HDx can increase clearance of κFLC and λFLC, whereas it is associated with reduced elimination of *β*2-microglobulin compared with HDF. The ability of HDx to remove small molecules is comparable to that of HDF.

In this meta-analysis, albumin removal with HDx was significantly greater than that with HD, leading to a decrease in the serum albumin concentration in the HDx group. With extended follow-up times however, the difference in predialysis serum albumin between the HDx group and the HD group gradually decreased. Hypoalbuminemia is associated with a poor prognosis in ESKD patients. Studies suggest that less than a 5% variation in serum albumin has no clinical significance [29,30]. Cho et al. [17] investigated whether using an MCO dialyzer for up to 12 months could keep serum albumin steady. They reported that after applying HDx for the first 2 months serum albumin decreased from baseline, but there was no significant decrease in serum albumin during the 12-month observation period. Weiner et al. [20] also reported that in the first 2 months of the study the HDx group had slightly lower predialysis serum albumin than the sHD group. After 24 weeks however, predialysis serum albumin levels did not differ significantly between the two groups. There was no significant difference in albumin loss in dialysate between the HDx and HDF groups. In the aforementioned trial reported by García-Prieto et al. [13], albumin leakage in the HDx group was significantly lower than that in the HDF group. In that trial, HDF was performed using an FX CorDiax 1000 dialyzer, with a bigger effective surface area and bigger UF coefficient, and reached higher convective volumes compared with Kirsch et al.’s study [1]. They measured albumin concentration in spent dialysate and multiplied the concentration at a certain timepoint with the flow rate at that same timepoint. If they used the flow rate of dialysate only (neglecting the convective UF rate) the calculated albumin loss would be falsely low for FX; whereas if they used the combined dialysate and convective UF flow rate the resulting albumin loss would be correct. The striking result was that albumin loss for FX was larger (not smaller) than that of MCO. A sensitivity analysis was performed in which that trial was excluded, and the results remained unchanged. In Kim et al.’s [31] trial albumin leakage in HDx was greater than predilution online HDF, but RR for albumin in HDF and MCO HD did not differ significantly. A sensitivity analysis was performed in which that trial was excluded, and the results remained unchanged. The current meta-analysis suggests that HDx can increase the clearance of κFLC and λFLC, whereas it does not increase the loss of albumin.

This meta-analysis was the first to evaluate the efficacy and safety of HDx compared to HD and HDF. The analysis had some limitations. First, most of the trials included only reported short-term results, thus we were unable to determine the long-term efficacy and possible adverse reactions associated with HDx. Second, different studies use different dialyzers for HD or HDF, which may have affected the results of the analysis. Third, the ultrafiltration values and the convective volume were different in different studies, which may have affected the results of the analysis. Fourth, this study was not registered. Lastly, the methods used to classify studies as high-quality may have been relatively lenient, and other researchers may have selected different definitions of study quality.

## Conclusion

HDx is superior to high-flux HD for the clearance of middle-sized and larger molecules. HDx can increase clearance of κFLC and λFLC, compared with HDF. There is no significant albumin loss in HDx treatment compared to HDF. It could be an alternative for patients in whom it is not possible to perform HDF. More randomized controlled trials are needed to determine the long-term safety and efficiency of HDx.

## Supplementary Material

Supplemental Material

Supplemental Material

Supplemental Material

Supplemental Material

## Data Availability

All datasets analyzed in this systematic review are referenced in the manuscript and additional files.

## Abbreviations

HDx: expanded hemodialysis
HD: hemodialysis
HDF: hemodiafiltration
CKD: chronic kidney disease
κFLC: kappa free light chains
λFLC: and lambda free light chains
SMD: standard mean difference
CI: 95% confidence
RR: Risk ratio
ESKD: End-stage renal disease
MCO: Medium cutoff dialyzers.
