# Supplementary material for: Efficacy and safety of expanded hemodialysis in hemodialysis patients: a meta-analysis and systematic review
Source: Ren Fail. 2022 Mar 28;44(1):541–50. doi: 10.1080/0886022X.2022.2048855 (PMC8967190; doi:10.1080/0886022X.2022.2048855)
Supplement: Supplemental Material [file IRNF_A_2048855_SM5107.pdf]

Search strategy

Search terms in Embase:

#1 'hemodialysis'/exp OR hemodialysis OR 'haemodialysis'/exp OR haemodialysis OR dialyzer* OR dialyser* OR membrane*

#2 theranova OR mco OR 'medium cut-off' OR 'mid cut-off' OR 'medium cut off' OR 'mid cut off' OR 'medium-cut off' OR 'mid-cut off' OR 'medium cutoff' OR 'mid cutoff' OR 'mco-hd' OR 'mco hd' OR 'expanded hemodialysis' OR 'expanded hd' OR 'hdx'/exp OR 'hdx'

#3 revaclear OR 'hd-c4' OR 'hd c4' OR 'hdc4'

#4 #1 AND #2 /py

#5 (#3 OR #4)

Cochrane Library

MeSH descriptor: [Renal Dialysis] explode all trees

#2 (hemodialysis) OR (dialyzer) OR ("dialyser") OR (membrane) (Word variations have been searched)

#3 #1 OR #2

#4 (Theranova) OR (MCO) OR (Medium cut-off) OR (MCO-HD) OR (Expanded hemodialysis) (Word variations have been searched)

#5 #3 AND #4

#6 (Revaclear) OR (HD-C4) OR (HDC4) (Word variations have been searched) 16

#7 #5 OR #6
