# Supplementary material for: Efficacy and safety of expanded hemodialysis in hemodialysis patients: a meta-analysis and systematic review
Source: Ren Fail. 2022 Mar 28;44(1):541–50. doi: 10.1080/0886022X.2022.2048855 (PMC8967190; doi:10.1080/0886022X.2022.2048855)

Funnel plot showing publication bias based on reduction rate of β2-microglobulin

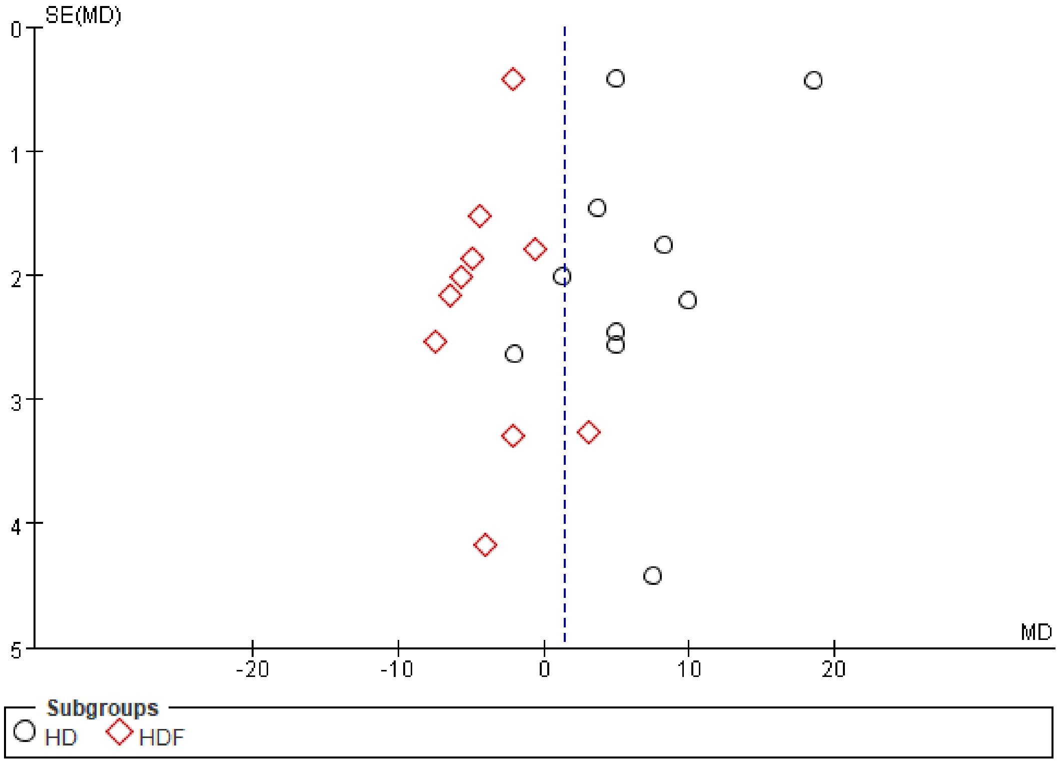

Funnel plot showing publication bias based on reduction rate of λFLC

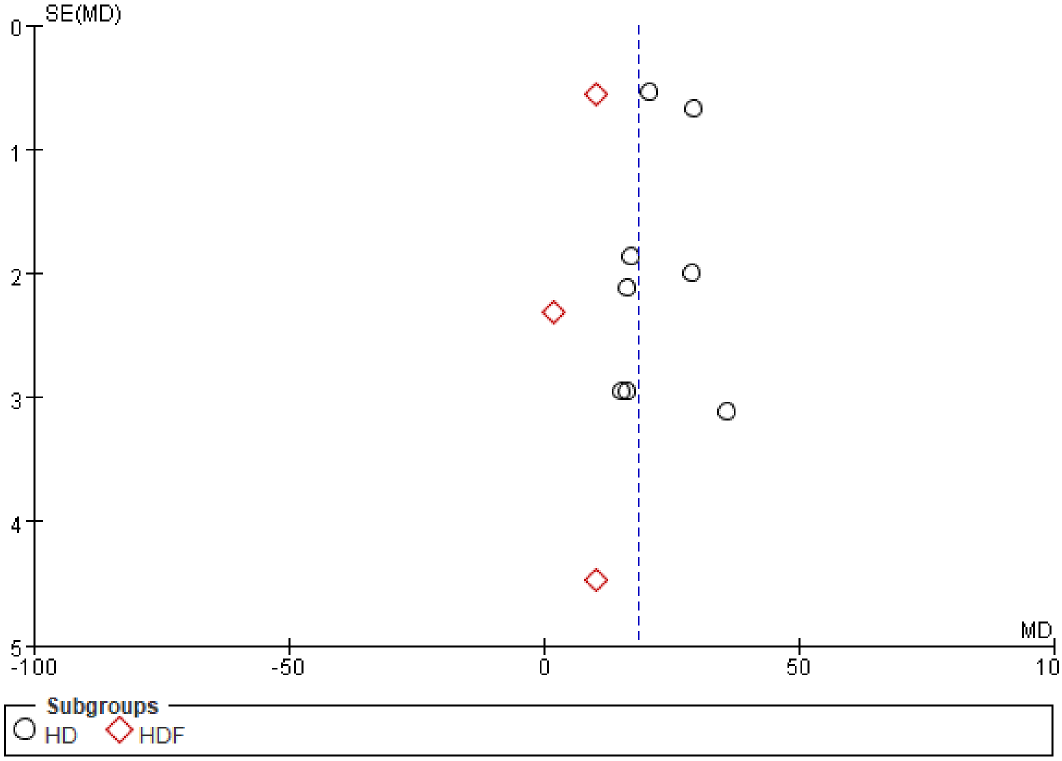

Funnel plot showing publication bias based on reduction rate of κFLC

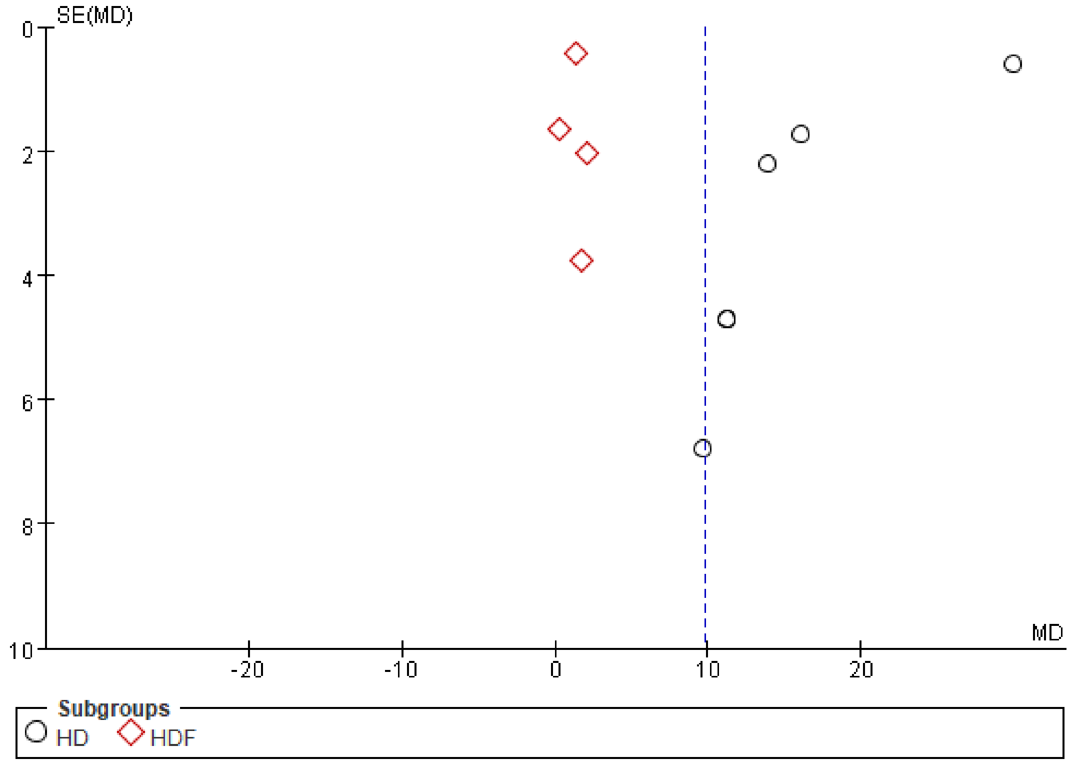

Supplement: Supplemental Material [file IRNF_A_2048855_SM5103.pdf]
