# Supplementary material for: Efficacy and safety of expanded hemodialysis in hemodialysis patients: a meta-analysis and systematic review
Source: Ren Fail. 2022 Mar 28;44(1):541–50. doi: 10.1080/0886022X.2022.2048855 (PMC8967190; doi:10.1080/0886022X.2022.2048855)
Supplement: Supplemental Material [file IRNF_A_2048855_SM5091.pdf]

Supplement Table 2: residual renal function in corelated studies

Studies		Residual renal function	HDx	HD/HDF
Belmouaz, 2019	cross-over study	urine output, < 200ml/d	19（95）	19（95）
Cho, NJ, 2019	-	lack data on residual renal functions	-	-
Cozzolino， 2021	cross-over study	lack data on residual renal functions	-	-
Lim, 2020	-	urine output	75.8±216.9	147.2 ±366.3
Sevinc, 2020	cross-over study	Urine output	0, (0-200)	0(0-37.5)
Weiner, 2020	-	No data on residual kidney function	-	-
Yeter, 2020	-	Urine output > 100ml, n（%）	5（34）	4（27）
García-Prieto,A, 2018	-	All had no renal residual function	0	0
Zickler, 2017		urine output < 500ml, n（%）	16	14
Arrascue, 2020	-	urine output	424±523.99	371±550.68
Cordeiro,2019	cross-over study	No data on residual kidney function	-	-
